# Supplementary material for: Monitoring, surveillance, antimicrobial resistance and genetic diversity analysis of non-typhoidal Salmonella in South Africa from 1960–2023 from animal and animal products
Source: PLoS One. 2025 Aug 1;20(8):e0329061. doi: 10.1371/journal.pone.0329061 (PMC12316224; doi:10.1371/journal.pone.0329061)
Supplement: Attachment — Submitted filename: Response to reviewers.docx [file pone.0329061.s003.docx]

|  | **Reviewer # 1 comments** | **Response** |
| --- | --- | --- |
|  | The manuscript goes into extreme detail regarding the predominant serotypes and AMR and much of this can be combined into a single argument, including the tables of resistance, resistant genes and serotypes, shortening the manuscript, especially as much of the data can be presented in figures/tables. | Thank you for your feedback. While we understand the suggestion to streamline the manuscript, we believe that the detailed discussion of predominant serotypes, antibiotic resistance (AMR), and their associated resistance genes is crucial for providing comprehensive insights into the study's findings. Each section offers unique contributions to the understanding of the complex relationships between Salmonella serotypes and antimicrobial resistance profiles, and combining these aspects may compromise the clarity and depth of the analysis.  Furthermore, while we agree that data can be effectively presented in figures and tables, the narrative explanation of the findings is necessary to provide context, interpretation, and highlight the significance of the results. The current structure aims to balance data presentation with in-depth analysis, ensuring that readers can appreciate both the broader trends and the nuances of the study.  We believe this approach enhances the manuscript’s value, and we are open to any suggestions for further conciseness without sacrificing the integrity of the information presented. |
| Line 206 | The authors have alluded to the source of the isolates - but have not provided any information on source attribution. Given the importance of this in NTS management, the authors should analyse which serotypes are associated with which food animals and include these data in the manuscript. | Kindly refer to line 249 -307. The author provide the link between the major serotypes to the source of isolation. |
| Line 211-215 | The authors refer to "Group OMD as the 8th most common serotype. This is incorrect. S. Gallinarum is the 8th most common serotype - the data needs to be reanalyzed with this in mind because OMD represents a collection of different serotypes, that agglutinate in group specific antisera. | The data has been reanalyzed to reflect S. Gallinarum as the eighth most common serotype, and **Group OMD** has been removed throughout the manuscript wherever prevalence was described or discussed. |
| Line 220-223 | Figure 1 - 3 are presented separately, but can be collapsed into a single figure reflecting the 8 most common serotypes (excluding OMD); the same applies to figures 4 and 5. | Figure 1–3 have been merged into a single figure, now referred to as “Figure 1, titled:  "Distribution of (a) total isolates, S. Typhimurium, S. Heidelberg, S. Enteritidis, and S. Minnesota, and (b) S. Dublin, S. Bovismorbificans, S. Infantis, and S. Gallinarum across six 10-year study periods." |
| Line 253-260 | Table 3 should be revised to exclude OMD as a serotype. | Table 3 has been renamed Table 4 and revised to exclude OMD as a serotype. |
|  | The authors, for convenience I suspect, divided their data into 13-year periods. I find this artificial and it obstructs from comparing these data to similar data that may be published elsewhere. They should rather use 5 - year or at least 10-year periods, and highlight that before 1960 and after 2020, the data are not representative of a full five years/decade. | The data has been analyzed in 10-year periods from lines 251 to 254, and all subsequent analyses followed this timeframe. Corrections have been made throughout the manuscript accordingly. |
|  | The authors need to completely revise the referencing. The numbers in the text do not correspond with the references which are presented alphabetically. | Thank you for the comment. The authors have revised the references accordingly |
|  | The authors should ensure that they have correctly italicised the genes referred to in the text | The authors have italicised all genes throughout the document |
|  | 3. Line 76 to 77: Beta-lactamase is not an antibiotic - it is an enzyme that breaks down BETA-lactam antibiotics | Thank you and that’s correct. The statement has been deleted. |
|  | Lines 119 - 121: this does not make sense and should be revised | The sentence has been revised in line 120-122 and reads as follows “In South Africa, the Animal Diseases and Parasites Act (1956) mandates that S. Enteritidis isolated from all fowls, including poultry and ostriches, must be reported to the state authority. |
|  | Please include the correct website address at line 158 - 159, instead of this version? | Corrected accordingly |
|  | The PCR methodology does not need to be described in full - the authors can state "as previously described and provide a reference. | Thank you for the comment. The PCR method was corrected as advised. |
|  | Line 473 ssspH1 should be corrected to sspH | Corrected accordingly |
|  | Line 546 & 565 - State the name of the author of each publication, not just the (incorrect) reference numbers [27] and [31]. This rule applies throughout | Corrected accordingly |
|  | Line 654. Delete the comma after patterns to give this sense | Comma deleted as advised |
|  | Line 661 - 662. Serotype severity does not differ between hosts, the severity of disease due to different serotypes does | The statement has been deleted |
|  | Lines 676 - 681 refer to antimicrobial use in chickens, but what is the potential role of environmental contamination in selecting for resistance? | Kindly refer to line 679 -689 where the authors added the contribution of environmental contamination. |
|  | Lines 701 - 708. Some of the isolates analysed go back to the 1950s. Can the authors be confident that their isolates did not lose virulence characteristics o | Its correct that bacteria can loss virulence characteristics over time particular if there are stored under unfavarable conditions for the long time. The authors acknowledge this and have responded to it in line 710-714 |
|  |  |  |
|  |  |  |
|  | **Reviewer # 2 comments** | **Response** |
|  | Line 562 states “… in an earlier study by [27].” Please add the name(s) of the author(s) being cited. | Corrected accordingly |
|  | Line 564 says “[31] provided the evidence …” Please edit it as requested above. | Corrected accordingly |
|  | Line 588 should start as a new sentence i.e. “On the grounds of these findings, …”. | Corrected as advised |
|  | Lines 631 to 632 are incomplete. “The resistance of piperacillin-tazobactam and ceftriaxone reported in this study was surprising since these antibiotics are registered in SA for human.” Human what? | Corrected accordingly |
|  | **Reviewer # 2 comments** | **Response** |
| Line 248-251 | While the total number of NTS isolates (1,028) is mentioned in the abstract, this detail is missing from the Materials and Methods section. It would be beneficial for the authors to specify how many isolates were obtained from animals, the environment, and food products, respectively. Additionally, it is unclear if there are differences in the types of Salmonella serovars detected across these sources. Including this information in a table would greatly enhance clarity and provide readers with a more comprehensive understanding of the study's findings. Although the association among antibiotic resistance, antibiotic resistance genes, and the sample source was mentioned. | The information on the source of isolation and its link to the major serotypes has been added as part of **Table 2**. |
