# Supplementary material for: Monitoring, surveillance, antimicrobial resistance and genetic diversity analysis of non-typhoidal Salmonella in South Africa from 1960–2023 from animal and animal products
Source: PLoS One. 2025 Aug 1;20(8):e0329061. doi: 10.1371/journal.pone.0329061 (PMC12316224; doi:10.1371/journal.pone.0329061)
Supplement: Attachment — Submitted filename: REVISED Respond to reviewer.docx [file pone.0329061.s004.docx]

**Journal Requirements:**

**All the responses in this rebuttal letter, including the corresponding line numbers, refer to the revised manuscript with track changes**

Comment 1:	Please review your reference list to ensure that it is complete and correct. If you have cited papers that have been retracted, please include the rationale for doing so in the manuscript text, or remove these references and replace them with relevant current references. Any changes to the reference list should be mentioned in the rebuttal letter that accompanies your revised manuscript. If you need to cite a retracted article, indicate the article’s retracted status in the References list and also include a citation and full reference for the retraction notice.

Respond 1:	We have reviewed the status of the papers listed in our references, specifically checking for any retraction notices. As of now, none of the references provided are flagged as retracted in major databases such as Retraction Watch, PubMed, or on the publishers’ websites. If the journal has identified any such papers, kindly provide us with the relevant details, and we will be happy to address the matter promptly.

**Additional Editor Comments:**

In addition to addressing the reviewers' comments, kindly fix the following issues:

Comment 2:	Please ignore reviewer 2's comment to change "virulence" to "virulent" and also in line 494 "virulence genes" to "virulent genes."

Respond 2: Duly noted, the authors didn’t change suggested by the editors

Comment 3. The pages of the revised manuscript should be numbered.

Respond 3:	Corrected

Comment 4. Kindly reduce the introduction section significantly to a maximum of two pages.

Respond 4:	Reduced to the required pages

Comment 5.	Summarize the text in lines 371-427 with appropriate tables. If this has been done already, kindly delete the text to avoid redundancy, as this manuscript is way too long. The information in lines 429-460 has been represented in figure 2'. Please reduce the text to a few sentences highlighting key results to avoid duplication of information already captured and redundancy.

Respond 5:	Deleted. The text has reduced in line 386 – 399. Please note that redundancy from 404 to 444 has been deleted.

Comment 6:	Summarize the text in lines 521-553 with a table and reduce the text significantly, highlighting only important results in a few sentences.

Respond 6:	The results are summarized in Table 6, while the findings related to virulence genes are presented only in paragraph form.

Comment 7.	Please reduce the discussion section significantly to a maximum of 3 pages discussing only major study results. Move the limitations to the last paragraph of the discussion section. Please delete the subheading "Study limitations".

Respond 7: The subheading has been removed from line 797. Additionally, the discussion section has been substantially shortened to three and a half pages, including the paragraph on the study’s limitations.

**Comment 8: Reviewer #1:** While the authors have addressed all the comments, I believe that they should still add information in the text and in table 2 on source attribution. Rather than say that Salmonella was isolated from meat, they should specify the type of meat (i.e. chicken, pork beef etc) as this has implications for disease control. If these data were not captured, they should address this in the text and the limitations.

**Respond 9:**	Incomplete entries in laboratory records and sample submission forms prevented us from capturing all necessary data, limiting our ability to draw more precise conclusions from the metadata, particularly in relation to source tracking. This point has been addressed in line 777-778

**Reviewer #2:** Dear Authors,
Your efforts on this manuscript are acknowledged and commended. I’ll start by agreeing with Reviewer#1 that this manuscript could have been more compact and easier to follow without losing relevant details if you had used tables and graphs instead of lengthy prose.

Comment 10:	What is the rationale behind the years you chose to review, especially 1959? I expected to read that in your methodology but may have missed it.

Respond 11:	As there were only a few isolates from 1950, the author has removed them from the analysis.

Comment 12:	Line 41 - 87% of the virulent (not virulence) genes screened …

Respond 12:	Please ignore reviewer 2's comment to change "virulence" to "virulent" and also in line 494 "virulence genes" to "virulent genes."

Comment 13:	Line 43 -... resistance, driven by misuse (of what), …

Respond 13:	Corrected in line 44

Comment 14:	Lines 43 to 44 -... could alter serotype dynamics (remove the comma) and change (not changing) disease epidemiology

Respond 14:	Corrected in line 45

Comment 15:	Line 54 -... cause cross-infection between animals and humans [3]. (end your sentence here and begin another) 99% of human serotypes belong to …

Respond 15:	Corrected in line 55

Comment 16:	Line 73 -... along with at least (why “at least”) amoxicillin clavulanic acid ...

Respond 16:	The sentence has been removed as part of the manuscript reduction.

Comment 17:	Line 77 - … towards fluoroquinolones (remove the comma) and third-generation cephalosporins.

Respond 17:	The sentence has been removed as part of the manuscript reduction.

Comment 18:	Line 97 -... operational in SA (write in full at first mention with the abbreviation in parentheses) …

Respond 18:	Corrected in line111

Comment 19:	Line 103 - delete “virology” as it is already included in “microbiology”.

Respond 19:	The sentence has been removed as part of the manuscript reduction.

Comment 20:	Line 113 - “… in the 1930s from …” not from the 1930’s.

Respond 20:	Corrected 129 in line

Comment 21:	Line 494 - “virulent genes” not “virulence genes”

Thank you!
