# Supplementary material for: Monitoring, surveillance, antimicrobial resistance and genetic diversity analysis of non-typhoidal Salmonella in South Africa from 1960–2023 from animal and animal products
Source: PLoS One. 2025 Aug 1;20(8):e0329061. doi: 10.1371/journal.pone.0329061 (PMC12316224; doi:10.1371/journal.pone.0329061)
Supplement: Attachment — Submitted filename: Response_to_Reviewers_auresp_3.docx [file pone.0329061.s005.docx]

**A rebuttal letter**

**Comment 1:	Journal Requirements:** Please review your reference list to ensure that it is complete and correct. If you have cited papers that have been retracted, please include the rationale for doing so in the manuscript text or remove these references and replace them with relevant current references. Any changes to the reference list should be mentioned in the rebuttal letter that accompanies your revised manuscript. If you need to cite a retracted article, indicate the article’s retracted status in the References list and also include a citation and full reference for the retraction notice.

**Responds 1:**	Thank you for the comment. The references have been revised, corrected, and completed both in-text and in the bibliography. No retracted papers were cited in this study. Due to the reduction in the manuscript length, some references were removed, which reduced the overall number of citations.

**Comment 2:	Additional Editor Comments:** In addition to addressing the reviewers' comments, please move the **limitations** to the **last paragraph of the Discussion** and **remove the subtitle "Study Limitations."**

**Responds 2:**	The necessary correction has been implemented on page 27, line 569 of the revised manuscript.

**Comment 3:	Reviewer #1:** I have one small comment - DALRRD is now Department of Agriculture. While I accept the work was probably done before the name change, I think it is worthwhile mentioning this under the financial support, for readers who are unaware of this. The manuscript need not go out to review again but this small addition can be made to the editor's satisfaction.

**Responds 3:	The necessary correction has been implemented on** page 27, line 613 of the revised manuscript.

**Comment 4**:	If applicable, we recommend that you deposit your laboratory protocols in [protocols.io](http://protocols.io/) to enhance the reproducibility of your results. Protocols.io assigns your protocol its own identifier (DOI) so that it can be cited independently in the future. For instructions see: [https://journals.plos.org/plosone/s/submission-guidelines#loc-laboratory-protocols](https://track.editorialmanager.com/CL0/https:%2F%2Fjournals.plos.org%2Fplosone%2Fs%2Fsubmission-guidelines%23loc-laboratory-protocols/1/010f019753aaa4ca-520db0f2-835f-4105-91aa-0d7fa9248b19-000000/qn931obIgW1YiWGXRlIWjCyJLFoJqaSD2tWubxXzRbU=214). Additionally, PLOS ONE offers an option for publishing peer-reviewed Lab Protocol articles, which describe protocols hosted on [protocols.io](http://protocols.io/). Read more information on sharing protocols at [https://plos.org/protocols?utm_medium=editorial-email&utm_source=authorletters&utm_campaign=protocols](https://track.editorialmanager.com/CL0/https:%2F%2Fplos.org%2Fprotocols%3Futm_medium=editorial-email%26utm_source=authorletters%26utm_campaign=protocols/1/010f019753aaa4ca-520db0f2-835f-4105-91aa-0d7fa9248b19-000000/d3V8muK-fm2TwCCmlrwLkKOS0NCqEpnuqXEeS7IuVMo=214).

**Respond 4**:	Your recommendations are duly noted. However, our laboratory protocols are considered intellectual property of the organization and are reserved for commercial purposes.
